# Neighbourhood Socioeconomics Status Predicts Non-Cardiovascular Mortality in Cardiac Patients with Access to Universal Health Care

**DOI:** 10.1371/journal.pone.0004120

**Published:** 2009-01-07

**Authors:** Claire L. Heslop, Gregory E. Miller, John S. Hill

**Affiliations:** 1 Atherosclerosis Specialty Laboratory, James Hogg iCAPTURE Centre for Cardiovascular and Pulmonary Research, Providence Heart+Lung Institute, Department of Pathology and Laboratory Medicine, University of British Columbia-St. Paul's Hospital, Vancouver, Canada; 2 Department of Psychology, Faculty of Arts, University of British Columbia, Vancouver, Canada; University of Cape Town, South Africa

## Abstract

**Background:**

Although the Canadian health care system provides essential services to all residents, evidence suggests that socioeconomic gradients in disease outcomes still persist. The main objective of our study was to investigate whether mortality, from cardiovascular disease or other causes, varies by neighbourhood socioeconomic gradients in patients accessing the healthcare system for cardiovascular disease management.

**Methods and Findings:**

A cohort of 485 patients with angiographic evidence of coronary artery disease (CAD) and neighbourhood socioeconomic status information was followed for 13.3 years. Survival analyses were completed with adjustment for potentially confounding risk factors. There were 64 cases of cardiovascular mortality and 66 deaths from non-cardiovascular chronic diseases. No socioeconomic differentials in cardiovascular mortality were observed. However, lower neighbourhood employment, education, and median family income did predict an increased risk of mortality from non-cardiovascular chronic diseases. For each quintile decrease in neighbourhood socioeconomic status, non-cardiovascular mortality risk rose by 21–30%. Covariate-adjusted hazard ratios (95% confidence interval) for non-cardiovascular mortality were 1.21 (1.02–1.42), 1.21 (1.01–1.46), and 1.30 (1.06–1.60), for each quintile decrease in neighbourhood education, employment, and income, respectively. These patterns were primarily attributable to mortality from cancer. Estimated risks for mortality from cancer rose by 42% and 62% for each one quintile decrease in neighbourhood median income and employment rate, respectively. Although only baseline clinical information was collected and patient-level socioeconomic data were not available, our results suggest that environmental socioeconomic factors have a significant impact on CAD patient survival.

**Conclusions:**

Despite public health care access, CAD patients who reside in lower-socioeconomic neighbourhoods show increased vulnerability to non-cardiovascular chronic disease mortality, particularly in the domain of cancer. These findings prompt further research exploring mechanisms of neighbourhood effects on health, and ways they may be ameliorated.

## Introduction

Personal socioeconomic status (SES) is an influential determinant of prognosis in coronary artery disease (CAD). Patients who have lower family incomes, limited education, and work in lower-prestige occupations are 1.5–2.0 times more likely to die in the years following diagnosis of angina pectoris and myocardial infarction [1]–[7].

Recently, it has become evident that the SES of the neighborhood in which a patient resides also contributes to CAD outcomes, and does so above and beyond the effects of his/her personal SES [8], [9]. For example, in a sample of more than 51,000 Canadian patients hospitalized for acute myocardial infarction, Alter *et al.* found that each $10,000 decrease in median neighborhood income was associated with a 10% increase in all-cause mortality over one year [10]. Tonne and colleagues studied 3423 patients hospitalized for myocardial infarction in Worcester, Massachusetts, and reported a 1.5-fold increase in mortality among those living in educationally disadvantaged neighborhoods [11].

These studies focused on the outcome of all-cause mortality. Thus, it remains unclear whether patients from economically disadvantaged neighborhoods are dying from CAD versus other conditions. Additionally, although neighbourhood unemployment is associated with presence of CAD [12], effects of neighbourhood unemployment on survival in CAD patients have not yet been adequately characterized.

In this article, we draw upon a >13 year follow-up of 485 patients in British Columbia with angiographically documented CAD, and examine socioeconomic disparities in mortality. Our objective was to investigate variations in total mortality, cardiovascular mortality, and deaths from other chronic diseases, across neighbourhood SES gradients.

Research of this nature is becoming especially relevant to developed countries which have medical systems that provide universal access to most basic care, such as Canada. The distribution of personal income in Canada has widened, which has contributed to increases in neighbourhood inequality in urban areas [13]. The impact of this trend on health outcomes, in the context of the ongoing expansion of private healthcare services, has not yet been clearly characterized.

## Methods

### Ethics Statement

All patients gave written informed consent; this research was approved by the Research Ethics Board of St. Paul's Hospital, Vancouver.

### Objectives

We sought to investigate whether mortality, from cardiovascular disease or other causes, varies by neighbourhood socioeconomic gradients in a cohort of stable coronary artery disease patients who accessed the same universal healthcare system but resided in different neighbourhoods within British Columbia, Canada.

### Cohort Patients

This article reports on 485 patients (383 men and 102 women) who were referred for selective coronary angiography at two Vancouver teaching hospitals between 1993 and 1995. These patients are a subset of a larger cohort of 1019 selective coronary angiography patients recruited to study novel risk factors for CAD and cardiovascular outcomes. Previous biochemical and genetic analyses of this cohort by investigators in our research group have been reported elsewhere [14]–[16]. To be included in the current analyses, patients had to provide a home postal code within British Columbia, and have coronary angiography results indicating CAD. Of the 1019 patients, 780 had angiographic evidence of CAD. Patients who did not provide a postal code, or provided a home address outside of British Columbia, were excluded (n = 295), leaving 485 CAD patients eligible for this study.

A questionnaire regarding clinical and lifestyle variables was administered to every patient by a nurse or attending cardiologist. Information was obtained on smoking status (ever, current and never), and alcohol consumption (never, 1–5, 6–10, and >10 drinks per week). Patient body weight, height, and blood pressure were measured. Patient history of diabetes and hypertension were obtained by self-report. In analyses, body mass index (kg/m^2^) was treated as a continuous variable, and diabetes and hypertension were categorized as no (0) and yes (1). Ever and current smoking status were combined to compare effects to patients who had never smoked.

### Coronary Angiograms

Each angiogram was assessed semi-quantitatively by a cardiologist blinded to any experimental results. Each lesion was assessed for percent diameter stenosis rounded to the nearest 10%. Patients in this group had one or more lesions of ≥10% stenosis. This definition was chosen because there is increasing evidence that small plaques may contribute more than large plaques to future risk of cardiovascular morbidity and death [17], and we hoped to avoid misclassifying patients due to subjective differences around the 50% stenosis mark.

### Mortality Data

In May, 2008, patients' identifying data were linked with the British Columbia Vital Statistics database to determine whether they had died prior to the end of 2007, the latest data available. Deaths occurring outside the province of British Columbia were not identified, thus these patients were treated as censored in survival analyses. Underlying cause of death codes for deceased patients were provided according to the World Health Organization International Classification of Disease, 10th revision (ICD-10) [18]. Bridge coding from ICD-9 to ICD-10 codes was completed by British Columbia Vital Statistics Agency prior to data provision.

Categories employed by the Canadian Centre for Chronic Disease Prevention and Control [19] were used to identify cardiovascular and non-cardiovascular chronic disease deaths The advantage of this approach is that categories are specific enough to permit disease-specific analyses, but broad enough to minimize variations in mortality coding that may have occurred across the follow-up time. Cardiovascular causes of mortality included ICD-10 codes I00–99. Non-cardiovascular chronic disease deaths included causes due to cancers (Neoplasm ICD-10 C00–97), chronic respiratory diseases (Respiratory Disease ICD-10 J00–98 minus Pneumonia, Influenza, and Acute Bronchitis ICD-10 J10–28), diabetes (ICD-10 E10–E14), mental disorders (ICD-10 F00–F89 and G20–30) or other chronic disease deaths (Chronic Liver Diseases ICD-10 K70, K73–74, Chronic Renal Failure ICD-10 N17–19, and Musculoskeletal Diseases ICD-10 M00–99). Deaths from non-chronic disease causes were included only for analyses of all-cause mortality.

### Neighbourhood Socioeconomic Information

Socioeconomic status data were derived from postal code information provided by cohort patients at baseline. Using Statistics Canada's 2001 Census of the Population [20], Semi-Custom Area Profile data, we derived median family income, as well as rates of education (percent without high school education), and unemployment (percent of adults over 25 seeking work), for each patient's neighbourhood. For each SES category, patients were placed in one of five quintiles, according to their neighbourhood's standing relative to the 469 neighbourhoods in the province of British Columbia. Higher scores represent increasing quintiles of neighborhood socioeconomic deprivation or disadvantage. SES data were compiled by the Mapping Unit of the Human Early Learning Partnership (HELP) at the University of British Columbia [21] using neighbourhoods defined by local populations in collaboration with research group mapping teams, and SES information obtained from a customized disaggregation of Statistics Canada 2001 census data for the 469 neighbourhoods.

### Statistical Analysis

Data were analyzed using SPSS version 14, and R version 2.7.2. Relationships between baseline covariates and mortality were assessed using Mann-Whitney U-tests for continuous variables, Pearson chi-square tests for categorical variables, and Mantel-Haenszel tests for linear trends in scaled categorical variables. The magnitude of associations between neighborhood SES and CAD risk factors was assessed by Pearson correlations for continuous variables, and point biserial correlations for categorical variables.

To assess relations between SES disadvantages and mortality, a series of Cox regression survival analyses were completed. Each SES indicator was entered as a continuous variable in each model, without adjustment, and then following forced-entry covariate adjustment for potential confounding variables age, sex, body mass index (BMI), diabetes, smoking status, and alcohol consumption. Model covariates were chosen to represent factors that are associated with risk of mortality, and/or may vary with neighbourhood composition.

Separate models were estimated for all-cause mortality, cardiovascular mortality, and for non-cardiovascular chronic disease mortality. Models were also generated for mortality from cancer, but due to the reduced number of deaths from this cause, only age, sex, BMI, and smoking were included as covariates to avoid over-adjustment [22], [23]. SES indicators were also entered as categorical variables in covariate adjusted models to permit comparison across quintiles, with the highest level of SES (quintile 1) used as reference. Linearity across SES quintiles were also tested using repeated contrasts to compare each quintile except the first category to the quintile that precedes it. Validity of the proportional hazards assumption for the survival models was verified using Schoenfeld residuals correlated with time, and partial residual plots for all survival models and covariates [24].

To measure survival model improvements offered by SES indices, area under the curve (AUC) values from time-adjusted receiver operator characteristic curves were generated from covariate-adjusted Cox regression models [25], using nearest neighbour kernel smoothing [26]. Models were tested for goodness of fit using Hosmer Lemeshow (HL) tests, which report significance values of p≤0.05 for risk models with significant deviation from accurate calibration across a range of risk [27].

## Results

### Differentials between Surviving and Deceased CAD Patients

For the current study we focused on 485 patients who had valid postal code data and angiographic evidence of CAD. Of these patients, there were 148 total cases of mortality, 64 of which were attributed to cardiovascular causes, and 66 of which were non-CAD chronic disease deaths. There were also 18 cases of deaths not caused by chronic diseases. Average and total follow-up times were 11.1 and 13.3 years.

The group of CAD patients represented all SES quintiles, with roughly equal numbers of patients in each of the 5 quintiles of income, education, and unemployment (Table 1). Table 2 displays patient baseline medical and risk factor covariate characteristics. At recruitment, the mean age of the patients was 61 years. The study group was 79% male, 72% were current or former smokers, and 19% had diabetes. Eighty-one percent of the patients reported European descent, with another 10% reporting either Chinese or South Asian ancestry.

**Table 1:** Baseline Numbers of Stable CAD Patients (n = 485) and Total Cumulative Mortality Rates after 13.3 Years Follow-Up Time Across Quintiles of Neighbourhood Socioeconomic Indices for Education, Unemployment and Median Family Income

Neighbourhood SES Category	Quintile (1 = high SES 5 = low SES)	CAD Patients (n)	Proportion (%)	Deceased (n)	Total Cumulative Mortality Rate (%)	p-value*
Education	1	146	30%	37	25%	0.06
	2	86	18%	38	32%	
	3	65	13%	17	26%	
	4	93	19%	30	32%	
	5	95	20%	36	38%	
Unemployment	1	63	13%	17	27%	0.26
	2	114	24%	33	29%	
	3	129	27%	41	32%	
	4	91	19%	24	26%	
	5	88	18%	33	38%	
Median Family Income	1	105	22%	29	28%	0.10
	2	86	18%	21	24%	
	3	131	27%	45	34%	
	4	76	16%	18	28%	
	5	87	18%	35	24%	

A review of angiography reports for this cohort suggested that approximately 90% of the CAD patients presented with stable disease, and the remaining patients presented with acute coronary syndromes (data not shown). As Table 2 shows, patients who were older at study entry were more likely to die over the follow-up, as were patients with diabetes. No other significant differences were observed among baseline variables. Missing response rates were 5% for smoking, 2.5% for alcohol consumption, and 0–2% for all other variables.

**Table 2:** Baseline Variable Means±Standard Deviations and Numbers (%) for CAD Patients (n = 485) and CAD Patients Alive or Deceased After 13.3 Years Follow-up Time

Variable	Categories or Units	All CAD Patients	Alive	Deceased	p-value*
Age	Years	61.0±10.5	59.3±10.1	64.9±10.3	<0.01
Sex	Male	383 (79)	270 (71)	113 (29)	0.35
	Female	102 (21)	67 (66)	35 (34)	
Ethnicity	European	392 (81)	266 (68)	126 (32)	0.09
	Chinese or South Asian	48 (10)	40 (83)	8 (17)	
	Other	45 (9)	31 (69)	14 (31)	
Smoking status	Never	122 (25)	91 (75)	31 (25)	0.16
	Ever or current	348 (72)	236 (68)	112 (32)	
Hypertension	No	183 (40)	193 (71)	80 (29)	0.67
	Yes	273 (60)	126 (69)	57 (31)	
Diabetes	No	393 (81)	284 (72)	109 (28)	<0.01
	Yes	92 (19)	53 (58)	39 (42)	
Alcohol consumption (drinks per week)	Never	118 (24)	86 (73)	32 (32)	0.81
	Occasional (1–5)	268 (55)	183 (68)	85 (32)	
	Moderate (6–10)	75 (15)	53 (71)	22 (29)	
	Heavy (>10)	12 (3)	9 (75)	3 (25)	
BMI	kg/m2	28.1±4.5	27.8±4.1	28.7±5.3	0.08

* p≤0.05 for differences in baseline variables between alive and deceased patients, from Mann-Whitney U tests and Pearson chi-square tests for continuous and categorical variables, respectively, and Manzel-Haenszel X^2^ tests for alcohol consumption.

CAD = coronary artery disease; BMI = body mass index

### Relationships between Neighbourhood SES and Patient Characteristics

Quintiles of SES variables were evenly distributed across cohort patients. Few significant relationships were observed between SES indices and patient characteristics. However, individuals with lower neighbourhood education levels were more likely to have a higher BMI (correlation of 0.09, p<0.05), and lower alcohol consumption (correlation of 0.11, p = 0.02). No other significant correlations were observed.

### Survival Analyses

The first wave of analyses tested for differences in all-cause mortality according to neighborhood disadvantage among individuals with CAD. There was no consistent effect of education, unemployment or family income on mortality. The unadjusted hazard ratios (95% confidence intervals (CI), significance value) were 1.11 (1.00–1.23, p = 0.05) for education, 1.08 (0.95–1.22, p = 0.24) for unemployment, and 1.10 (0.98–1.24, p = 0.10) for income.

The next wave of analyses examined SES disparities in cause-specific mortality. None of the indices of neighborhood disadvantage was associated with mortality from cardiovascular disease over the follow-up time. Unadjusted hazard ratios (95% CI, and significance values) were 1.03 (0.87–1.21, p = 0.75) for education, 0.96 (0.79–1.17, p = 0.69) for unemployment, and 1.02 (0.86–1.23, p = 0.80) for income.

**Figure 1:**
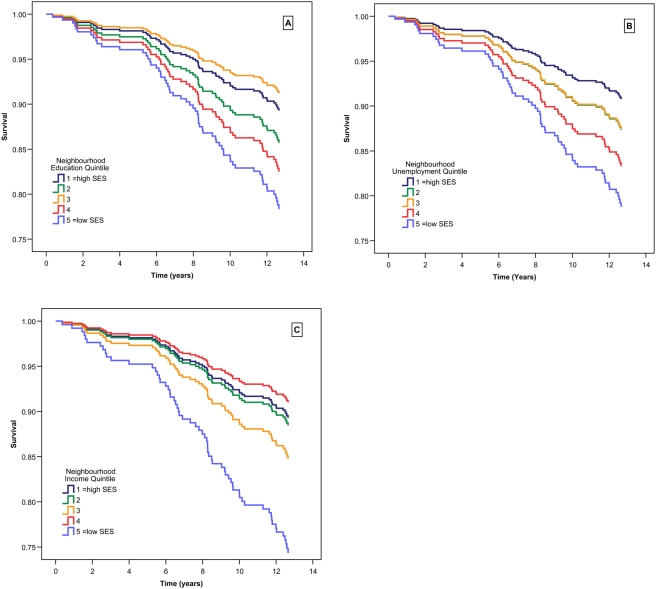
Survival curves for non-cardiovascular chronic disease mortality by neighbourhood socioeconomic quintiles for 485 coronary artery disease patients after 13.3 years follow-up time. Quintiles of A) education, B) unemployment, and C) median family income are relative to 469 neighbourhoods in the province of British Columbia mapped from the Human Early Learning Partnership mapping project, with neighbourhood socioeconomic data derived from Statistics Canada 2001 census. Higher scores represent increasing deprivation or disadvantage. Survival curves are derived from Cox regression survival analyses with adjustment for age, sex, body mass index, diabetes, smoking history, and alcohol consumption.

However, there were significant disparities in non-cardiovascular chronic disease mortality rates by SES. Each one quintile increase in neighbourhood unemployment was associated with a 30% greater risk of non-cardiovascular chronic disease death, following adjustment for risk factors (age, gender, BMI, diabetes, smoking, and alcohol consumption). Quintiles of median family income and neighbourhood education levels were both associated with 21% increases in risk, following adjustment for the same risk factors. Linearity of SES effects for education, income and unemployment were confirmed in covariate-adjusted models as described above for all neighbourhood SES indices (p<0.05 for all SES measures). Results from unadjusted survival models, and forced-entry covariate adjusted survival models, are displayed in Table 3. Survival curves from covariate adjusted models are depicted graphically in the panels of Figure 1.

**Table 3:** Hazard Ratios (95% Confidence Intervals) from Survival Analyses for Non-Cardiovascular Chronic Disease Mortality by Neighbourhood SES Category Quintiles for CAD Patients (n = 485) After 13.3 Years Follow-Up Time

	Covariates in Models	Education	Unemployment	Median Family Income
Survival model without covariates	SES quintile	1.20 (1.02–1.42)*	1.23 (1.01–1.50)*	1.24 (1.03–1.48)*
Survival model with covariates: Step 1	Age (years)	1.07 (1.04–1.10) †	1.07 (1.04–1.11) †	1.07 (1.04–1.10) †
	Male sex	0.92 (0.49–1.71)	0.95 (0.51–1.77)	0.97 (0.52–1.82)
	BMI	1.02 (0.96–1.08)	1.02 (0.97–1.08)	1.03 (0.97–1.08)
	Diabetes	1.76 (0.99–3.13)	1.92 (1.08–3.43)*	1.82 (1.03–3.22)*
	Smoking (ever or current)	2.21 (1.13–4.31)*	2.12 (1.08–4.13)*	2.13 (1.10–1.16)*
	Alcohol consumption	1.06 (0.68–1.39)	0.89 (0.61–1.31)	0.93 (0.63–1.36)
Step 2	SES quintile	1.20 (1.02–1.42)*	1.30 (1.06–1.60)*	1.21 (1.01–1.46)*

Hazard ratios, 95% confidence intervals, and significance values for each quintile increase in SES indices are given from Cox regression models for risk of non-cardiovascular chronic disease mortality. Covariates listed were force-entered in adjusted Cox regression models

* p≤0.05 ^†^p≤0.01 SES = socioeconomic status; BMI = body mass index

Time-adjusted AUC values for non-cardiovascular chronic disease mortality with and without SES indicators were generated from adjusted Cox regression survival analyses. AUC values obtained were 0.600 for covariates age, sex, BMI, diabetes, smoking, and alcohol consumption without SES indicators. The AUC values improved to 0.712, 0.715, and 0.720 for the model with addition of indices for education, unemployment, and income, respectively.

HL tests of model calibration, for which p≤0.05 indicates poor model calibration across a range of risk, show good risk model fit for prediction of non-cardiovascular mortality for the covariate-adjusted adjusted models tested above. Models including ; ; with SES variables education, unemployment and income, yielded HL chi-square test statistics of 9.17 (p = 0.33), 8.16 (p = 0.42), and 6.94 (p = 0.54), respectively, and the basic model statistic was 5.71 (p = 0.68).

### Disease Specific Survival Analyses

To discern which causes of death were underlying these associations, we carried out secondary analyses of mortality from specific non-cardiovascular chronic diseases. Cancer was the most common cause of death in this category, with 31 patients (6.4% of the cohort) having died from neoplastic diseases. We did not carry out further analyses for death from other types of chronic diseases, due to insufficient numbers in each category.

**Figure 2:**
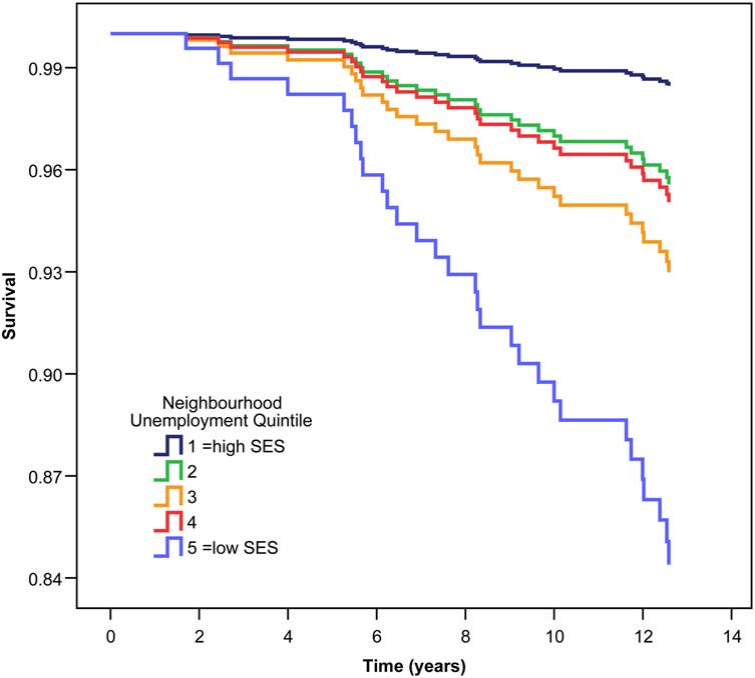
Survival curve for cancer mortality by quintile of neighbourhood unemployment for 485 coronary artery disease patients after 13.3 years follow-up time. Quintiles of neighbourhood unemployment are relative to 469 neighbourhoods in the province of British Columbia mapped from the Human Early Learning Partnership mapping project, with unemployment data derived from Statistics Canada 2001 census. Higher quintiles represent increasing levels of neighbourhood unemployment. Survival curves are derived from Cox regression survival analyses with adjustment for age, sex, body mass index, and smoking history.

As Table 4 shows, Cox survival analyses revealed significant neighborhood disparities in cancer mortality for unemployment and family income. These relationships withstood adjustment for the covariates age, sex, BMI, and smoking history. For neighbourhood unemployment, risk of death from neoplastic causes increased 62% per quintile (p<0.01). This relationship is displayed graphically in Figure 2. A parallel finding was evident for median family income, with a 42% increase in cancer mortality per quintile (p = 0.01). Linearity of SES effects for unemployment and income were confirmed in covariate-adjusted models (p = 0.01 for unemployment, p = 0.04 for income).

**Table 4:** Hazard Ratios (95% Confidence Intervals) from Survival Analyses for Cancer Deaths by Neighbourhood SES Category Quintiles for CAD Patients (n = 485) After 13.3 Years Follow-Up Time

	Covariates in Models	Education	Unemployment	Median Family Income
Survival model without covariates	SES quintile	1.11 (0.88–1.41)	1.56 (1.16–2.11) †	1.40 (1.07–1.83)*
Survival model with covariates: Step 1	Age (years)	1.05 (1.01–1.09)*	1.05 (1.01–1.10)*	1.04 (1.00–1.09)*
	Male sex	0.99 (0.40–2.43)	1.01 (0.41–2.48)	1.05 (0.43–2.59)
	BMI	0.97 (0.89–1.05)	0.97 (0.89–1.06)	0.97 (0.89–1.06)
	Smoking (ever or current)	3.25 (1.11–9.51)*	2.98 (1.02–8.76)*	3.19 (1.06–9.33)*
Step 2	SES quintile	1.18 (0.94–1.48)	1.62 (1.20–2.19) †	1.42 (1.09–1.84) †

Hazard ratios, 95% confidence intervals, and significance values for each quintile increase in SES indices are given from Cox regression models for risk of cancer mortality. Covariates listed were force-entered in adjusted Cox regression models.

* p≤0.05 ^†^p≤0.01 SES = socioeconomic status; BMI = body mass index

Comparing patients in the lowest versus highest quartiles of SES, there was a 10-fold disparity in cancer mortality from the highest to the lowest quintile of neighbourhood unemployment (95% CI 1.31–76.33, p = 0.03). There was a 5.2–fold increase in cancer mortality from the highest to lowest quintile of neighbourhood median family income (95% CI 1.47–18.5, p = 0.01). No significant relationship was observed for education.

Despite the limited number of covariates included, the AUC value for the cancer mortality model with age, sex, BMI and smoking, improved from 0.651 to 0.720 and 0.712 for SES indices of unemployment and income, respectively. HL tests indicated no significant deviation from model calibration with SES variables. Covariate-adjusted model HL X^2^ test statistics were 4.09 (p = 0.85), and became 9.17 (p = 0.33), 4.57 (p = 0.80), and 8.09 (p = 0.43) with the addition of indices for education, unemployment, and family income, respectively, confirming no loss of model calibration with the addition of SES indices.

## Discussion

We investigated whether neighbourhood socioeconomic conditions predicted mortality among 485 stable coronary artery disease patients living in British Columbia, after >13 years of follow-up time. Based on the extensive literature documenting social gradients in morbidity and mortality from CAD [1]–[7], [9], [10], we expected that patients with adverse neighbourhood socioeconomic conditions would be especially prone to cardiac death. However, we did not find a gradient in overall or cardiovascular mortality attributable to neighbourhood characteristics. Instead we observed a marked gradient in mortality from non-cardiovascular chronic diseases. For each quintile increase in neighbourhood SES deprivation, estimated risks for non-CAD chronic disease deaths increased between 21–30%, leading to an average 2.4-fold increase between highest and lowest neighbourhood SES quintiles. Although the number of cancer deaths were small, profound effects were observed for rates of cancer mortality; estimated risks for cancer death increased 42% and 62% for each quintile decrease in neighbourhood SES family income and employment, respectively.

Although it would not be appropriate to use neighbourhood SES solely to distinguish which CAD patients should be screened for other chronic diseases, we demonstrate that neighbourhood SES indices improved risk prediction for chronic disease mortality in these patients. Areas under time-adjusted receiver operator curves for non-cardiovascular and cancer deaths increased when neighbourhood SES was added to a risk model that included age, sex, BMI, diabetes, smoking, and alcohol consumption. Also, calibration tests well suited to assess cohorts of this size [28] show that good model calibration was achieved across range of risk for covariate-adjusted models.

Alongside numerous studies demonstrating increased incidence of CAD and cardiovascular death in neighbourhoods with lower SES, there is extensive literature documenting higher total mortality among CAD patients living in lower SES neighbourhoods. However, few studies have investigated which specific types of mortality are increased among CAD patients living in lower SES neighbourhoods. We distinguish cardiovascular mortality from deaths due to other chronic diseases, and add new information regarding the nature of these increased risks.

It is not clear why a social gradient in cardiovascular deaths did not emerge in our data. Two other studies have found that CAD patients from low SES neighborhoods are at increased all-cause mortality risk [10], [11]. These studies had much larger sample sizes than ours, so reduced statistical power may have masked these effects in our study. The patients in the other studies had recently been hospitalized for myocardial infarction, so these subjects may have had more advanced disease or been less medically stable in comparison to patients in our study. Also, patients in the other studies were drawn from many regional acute care centers, while our cohort patients were investigated by selective coronary angiography and treated in cardiology services at major teaching hospitals, thus similarities of care may have eliminated—or at least diminished—existing social disparities in prognosis. Disparities in care that associate with neighbourhood SES could explain why these studies observed associations between neighbourhood and cardiovascular mortality and we did not. But as we can presume care was cardiac-specific, it would not have had the same effects for other chronic diseases such as cancer. Future research is needed to explore this possibility, and to determine the applicability of our findings to other cardiac populations.

Our findings are consistent with published evidence showing a gradient in health across neighbourhoods, however it remains unclear whether our findings are attributable to community versus individual SES, as the former could simply be acting as a proxy measure for variations in the latter. Both of these factors predict mortality from a variety of causes, and in many cases they do so independently of one another [8], [9], [29], [30]. Our findings that non-CAD chronic disease death rates increase with neighbourhood SES deprivation in CAD patients suggests neighbourhood conditions may independently instigate longer term health effects which persist in the context of a medical system designed to provide care regardless of individual-level SES. Health status of residents of British Columbia do vary markedly according to where they live, with more geographically compact and populous areas of BC showing the best overall health status, and more sparely populated areas showing the worst [31]. British Columbians with lower incomes do show more frequent use of general practitioners and acute inpatient care, while higher income is associated with the greater use of specialist and surgical day care service [32]. Without having individual SES data for the patients in this sample, we cannot discriminate between these competing explanations for the findings.

Living in a lower-SES neighborhood may increase people's exposure to pollutants, infections, and carcinogens that contribute to the pathogenesis of chronic diseases such as cancer [33]. However, these exposures seem unlikely to be the primary mechanism at play here, because the social gradient in mortality we observed were fairly linear in nature. It is difficult to imagine that exposures are distributed in this fashion as well; i.e. that for each increment in neighborhood SES there is a corresponding reduction in contact with toxicants. Our analyses also controlled for key demographic characteristics and lifestyle variables, thus we believe these factors are unlikely to have played a major role.

Another possibility is that daily stress associated with living in an impoverished neighborhood takes a biological toll on the body. There is a roughly linear inverse association between social class and perceived stress [34], and persons residing in low-SES communities release higher levels of stress-related hormones, have more systemic inflammation, and are more likely to display features of metabolic syndrome [35]–[37]. These stress-related biological perturbations may accumulate over time in a manner that contributes to the development and/or progression of chronic diseases such as cancer [38], [39], and the findings of our study are consistent with this theory.

### Limitations

This study has limitations that need to be considered. Individual-level socioeconomic information was not collected at baseline. Thus, we are unable to take this data into account when evaluating neighbourhood socioeconomic effects, or to compare the magnitude of individual- versus neighbourhood-level socioeconomic gradients. Both patient self report and measurements at baseline were used to gather information about risk factors. These approaches are subject to recall bias and measurement error, respectively. Also, our participants were not asked about personal history of cancer at initial baseline assessment. Thus, despite other studies suggesting no significant difference in rates of cancer between higher and lower socioeconomic status neighbourhoods in Canada [40], we cannot correct for presence of neoplastic disease prior to study entry. The higher risk of neoplastic mortality observed among individuals in lower-SES neighbourhoods may be partly to lower utilization of diagnostic imaging, as has been documented elsewhere in Canada [41]. Unfortunately, we cannot ascertain how this factor contributes to our findings. It will be important for future research to include this information. Finally, this study is not sufficiently powered to investigate each cause of death separately. These limitations need to be considered in light of the project's strengths, including its prospective design, well-characterized patients, long follow-up time, and complete ascertainment of mortality.

Our findings suggest that even in a country with universal healthcare services, there are marked gradients in mortality according to neighbourhood. We demonstrate the predictive strength of neighbourhood SES above and beyond key modifiable and non-modifiable risk factors for mortality, and suggest that broader approaches to recognizing and addressing socioeconomic inequalities are needed. In order to achieve more equitable healthcare and overcome disparities in resources and access, we must create an improved appreciation of social and biological factors responsible for poor health outcomes, and develop the economic, behavioral, and biomedical interventions needed to ameliorate them.
